# Sturge–Weber syndrome coexisting with autosomal dominant polycystic kidney disease

**DOI:** 10.1007/s11255-012-0243-8

**Published:** 2012-07-25

**Authors:** Mariusz Niemczyk, Renata Niemczyk, Monika Gradzik, Stanisław Niemczyk, Dariusz Kęcik, Leszek Pączek

**Affiliations:** 1 Department of Immunology, Transplant Medicine and Internal Diseases, Medical University of Warsaw, Nowogrodzka 59, 02-006 Warsaw, Poland; 2 Department of Ophthalmology, Medical University of Warsaw, Warsaw, Poland; 3 1st Department of Radiology, Medical University of Warsaw, Warsaw, Poland; 4 Department of Internal Diseases, Nephrology, and Dialysis, Military Institute of Medicine, Warsaw, Poland

**Keywords:** Autosomal dominant polycystic kidney disease, Sturge–Weber syndrome, Complications, Phakomatoses

**Fig. 1:**
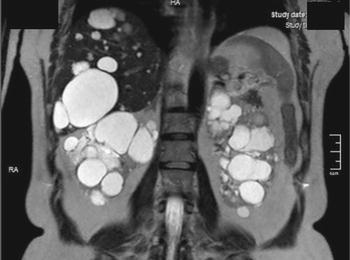
Polycystic kidneys in magnetic resonance imaging

**Fig. 2:**
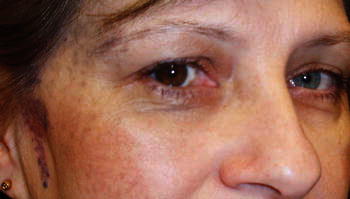
Typical features of Sturge–Weber syndrome include ill-defined, non-elevated cutaneous angioma, localized in the ophthalmic and maxillary distributions of the trigeminal nerve, also known as the port-wine stain, and heterochromia of the iris with hyperchromic iris and episcleral hemangiomas of the ipsilateral eye

**Fig. 3:**
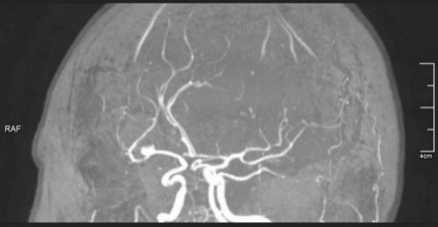
Magnetic resonance angiography of the intracranial arteries revealed an aneurysm 5 × 4 mm in the division of the right middle cerebral artery, which is a feature of autosomal dominant polycystic kidney disease

Editor,

In a 50-year-old white woman with stage 3 chronic kidney disease in a course of the autosomal dominant polycystic kidney disease (ADPKD), diagnosed with imaging examination of the abdominal cavity (Fig. 1) and arterial hypertension, Sturge–Weber syndrome (SWS) was diagnosed on the basis of cutaneous [1] and ophthalmologic [2] signs (Fig. 2). Both ADPKD and SWS increase the risk of stroke [3]: the former due to increased prevalence of intracranial aneurysms, and the latter due to leptomeningeal angiomas. Therefore, despite the absence of neurologic symptoms, magnetic resonance angiography of the intracranial arteries was performed, which revealed intracranial aneurysm (Fig. 3).

The diagnostic features of SWS include: (1) unilateral facial angioma, known as the port-wine stain, localized in the I, and, less often, in II, and III sensory distribution of the trigeminal nerve, and occasionally involving the neck and trunk, (2) ipsilateral leptomeningeal angiomatosis in the parietal–occipital lobe, and (3) congenital glaucoma in 30–70 % of cases. However, the manifestation of SWS is often partial or incomplete. Therefore, SWS is divided into 3 types: type I, known as classic SWS, with facial and leptomeningeal manifestations and possible glaucoma; type II, in which facial angioma is present, with possible glaucoma, but without intracranial disease; and type III, limited to leptomeningeal angioma [4, 5].

Absence of leptomeningeal angiomas led to the diagnosis of type II SWS in the reported case. Additionally to neurosurgical consultation, patient was referred to the ophthalmologist, as SWS may be connected to the risk of progressive vision loss of the eye ipsilateral to the skin changes due to glaucoma, or complications of diffuse choroidal hemangioma, such as cystoid macular edema, and exudative retinal detachment.

SWS belongs to a group of rare disorders known as phakomatoses. Some of them, like tuberous sclerosis and von Hippel–Linadu syndrome, may be associated with polycystic kidney disease [6]. However, until now, coexistence of SWS and ADPKD has never been reported. The connection between SWS and ADPKD also in our patient is unlikely, especially that three of her sisters have ADPKD, but not SWS.

Summarizing, a patient with morphological features of SWS should be examined for neurologic and ophthalmologic elements of the disease, which may lead to serious complications.
